# Insulin-producing cells could not mimic the physiological regulation of insulin secretion performed by pancreatic beta cells

**DOI:** 10.1186/1556-276X-8-90

**Published:** 2013-02-19

**Authors:** Qiping Shi, Simin Luo, Haiying Jia, Lie Feng, Xiaohua Lu, Lixin Zhou, Jiye Cai

**Affiliations:** 1 The First Affiliated Hospital, Jinan University, Guangzhou 510632, China; 2 Institute for Nano-Chemistry, Department of Chemistry, Jinan University, Guangzhou 510632, China

**Keywords:** Insulin-producing cells, Human normal pancreatic beta cells, Atomic force microscopy

## Abstract

**Objective:**

The aim of this study was to compare the difference between insulin-producing cells (IPCs) and normal human pancreatic beta cells both in physiological function and morphological features in cellular level.

**Methods:**

The levels of insulin secretion were measured by enzyme-linked immunosorbent assay. The insulin gene expression was determined by real-time quantitative polymerase chain reaction. The morphological features were detected by atomic force microscopy (AFM) and laser confocal scanning microscopy.

**Results:**

IPCs and normal human pancreatic beta cells were similar to each other under the observation in AFM with the porous structure features in the cytoplasm. Both number of membrane particle size and average roughness of normal human beta cells were higher than those of IPCs.

**Conclusions:**

Our results firstly revealed that the cellular ultrastructure of IPCs was closer to that of normal human pancreatic beta cells, but they still could not mimic the physiological regulation of insulin secretion performed by pancreatic beta cells.

## Background

Diabetes is caused by absolute or relatively insufficient insulin secretion. Hitherto, there is no cure for diabetes. Treatment with insulin prolongs survival and improves glycemic control, and current standard diabetes treatment regimens with insulin replacement remain away from ideal. Transplantation of either isolated islets or the whole pancreas provides another mode for insulin replacement [1] but is often accompanied by many undesirable side effects [2-4]. Insulin-producing cells (IPCs) from pluripotent stem cells offer the potential to treat diabetes by providing a source for injured pancreatic beta cells without the limitations of current therapeutic modalities. To be successful, yet, IPCs must possess physiologically appropriate regulation of insulin secretion [5,6], including sensing circulating glucose concentrations and secreting insulin in response to physiological glucose concentrations appropriately without risk of neoplastic transformation [7,8]. Nowadays, unresolved obstacles associated with differentiation of stem cells into IPCs include maturation of the insulin secretory pathways and mechanisms responsible for sensing ambient glucose concentrations as well as lack of sufficient development of the insulin processing machinery [9,10].

Atomic force microscopy (AFM) has been widely used in cell biology studies, especially of both cellular and subcellular structures and topographical morphology [11,12], because of its ability to image biological samples at nanometer resolutions. Differences in cell morphology can likely reveal the reason why there is great difference in cellular function. Thus, we compared the differences in morphology and function between normal human pancreatic beta cells and IPCs derived from human adipose-derived stem cells (hADSCs). Moreover, we examined the relationship between cell morphology and function. At the molecular level, we found that although IPCs had a similar distribution of membrane proteins to normal pancreatic beta cells, they still could not mimic the physiological regulation of insulin secretion performed by normal pancreatic beta cells. We propose that the difference in physiological function between these two kinds of cells is due to the difference in the nanostructure of their cell membranes.

## Methods

### Isolation and differentiation of MSCs from human adipose tissue

Human adipose tissue was obtained from four donors, two males and two females. Informed consent was obtained from participating donors according to procedures approved by the Ethics Committee at the Chinese Academy of Medical Sciences. Experiments were performed according to the ethical standards formulated in the Helsinki Declaration. The isolated and differentiated procedure was described by Shi et al. [13]. In order to authenticate the phenotypes of mesenchymal stem cells (MSCs), flow cytometric analysis of hADSCs was performed using antibodies for CD59, CD34, CD44, CD45, CD105, CD13, and HLA-DR (BD Biosciences, Franklin Lakes, NJ, USA).

### Culture of normal human pancreatic beta cells

Normal human pancreatic beta cells were obtained commercially (HUM-CELL-0058, Wuhan Pricells Biotechnology & Medicine Co., Ltd., Wuhan, China). Expansion medium contained MED-0001 and 5 ng/mL rhEGF, 5 μg/mL rhinsulin, 5 μg/mL transferrin, 10 nM T3, 1.0 μM epinephrine, 5 μg/mL hydrocortisone, 10% fetal bovine serum (all expansion media were from Wuhan Pricells Biotechnology & Medicine Co., Ltd.). The cells were cultured in complete medium in T-25 tissue culture flasks that have been coated with collagenase at 37°C in 5% CO_2_. Culture medium was changed every other day.

### Insulin assay

Both normal human pancreatic beta cells and IPCs were preincubated in Dulbecco's phosphate-buffered saline (D-PBS, without glucose), low-glucose Dulbecco's modified Eagle's medium (DMEM; 5.5 mM, Gibco, Grand Island, NY, USA), or high-glucose DMEM (25 mM, Gibco) for 1 h or 30 min. The buffers from six wells of cells were collected separately. The amount of insulin in the buffer of each well was determined by ultrasensitive insulin enzyme-linked immunosorbent assay (ELISA) and normalized by the number of cells in each well.

### Quantitative gene expression analysis

Total RNA was collected from cells using TRIzol reagent (Invitrogen, Carlsbad, CA, USA) and treated with DNase. Total RNA (1 μg) was analyzed by quantitative reverse transcription-polymerase chain reaction (qRT-PCR) in an ABI 7000 Real-Time PCR System (Applied Biosystems, Foster City, CA, USA) using the Sybr-Green primers. Real-time PCR was performed using a real-time PCR Taq core kit (Takara, Dalian, China). The reaction consisted of 50 μL, containing 25 μL Sybr-Green, 16 μL H_2_O, 5 μL cDNA, 2 μL sense primer (10 μM), and 2μL antisense primer (10 μM). The conditions were set in accordance with the manufacturer's protocol. Expression was calculated relative to glyceraldehyde-3-phosphate dehydrogenase (GAPDH). All primers were from Invitrogen (Table 1).

**Table 1:** Sequences of primers for real-time qRT-PCR

**Primer**	**Sense (5**^**′**^**-3**^**′**^**)**	**Antisense (5**^**′**^**-3**^**′**^**)**
Insulin	5^′^-GCAGCCTTTGTGAACCAACA-3^′^	5^′^-TTCCCCGCACACTAGGTAGAGA-3^′^

### Sample preparation for AFM

To detect the morphological changes of beta cells and IPCs before and after glucose stimulation, cells were separated into five groups: glucose-free culture medium group (D-PBS), 30-min low-glucose stimulation group, 1-h low-glucose stimulation group, 30-min high-glucose stimulation group, and 1-h high-glucose stimulation group. Cell samples were preincubated for 1 h or 30 min in D-PBS, low-glucose DMEM (Gibco), or high-glucose DMEM (Gibco). They were then washed in distilled water twice before being fixed with 2.5% glutaraldehyde for 20 min. The samples were washed in distilled water three times again, then air-dried for AFM scanning.

### AFM measurement

An Autoprobe CP AFM (Veeco, Plainview, NY, USA) was used in contact mode to detect the immobilized IPCs and normal human pancreatic beta cells at room temperature. Silicon nitride tips (UL20B, Park Scientific Instruments, Sunnyvale, CA, USA) were employed in all AFM measurements. An optical microscope was used to help select the desired cells and direct the position of the AFM tip. Single-cell imaging was repeated for six cells, and each cell was scanned for three times. All images were analyzed by the instrument-equipped software (Image Processing Software Version 2.1) to gain information on the topography. ‘Ra’ denotes the average roughness in the analytical area. All parameters were directly generated by the software IP2.1.

### LCSM and observation

Cells were fixed in 2.5% glutaraldehyde for 30 min, washed in PBS, and then permeabilized in 0.1% Triton X-100 at room temperature for 30 min. After, cells were washed in PBS thoroughly. Cells were then incubated with 1 μM phalloidin-rhodamine (Biotium, Inc., Hayward, CA, USA) at 4°C overnight to label F-actin. After several washes in PBS, the labeled cells were scanned by LCSM (510 Meta Duo Scan, Carl Zeiss, Oberkochen, Germany) using 545-nm (He-Ne) excitation. Emission was detected above 600 nm.

### Statistical analysis

All data were presented as mean values ± standard deviation taken from ten different cells. The morphologic parameters between the different groups were compared using *t* test (via SPSS 11). Differences with *P* values less than 0.05 were considered to be statistically significantly.

## Results

### Morphology and phenotypes of cultured hADSCs

Isolated hADSCs exhibited a spindle shape, began to appear in culture, and reached 90% confluence in about 10 to 12 days. The second passage of hADSCs expanded rapidly and developed a uniform morphology that resembled that of fibroblasts. FACS analysis of hADSCs at the third passage showed that these cultured cells were positive for CD13 (98.88%), CD44 (98.9%), CD59 (98.4%), and CD105 (71.24%). In addition, expression of HLA-DR (0.98%) was not detected. Furthermore, hADSCs exhibited low expression of hematopoietic lineage markers CD45 (1.03%) and CD34 (2.88%).

### Differentiation of IPCs

**Figure 1:**
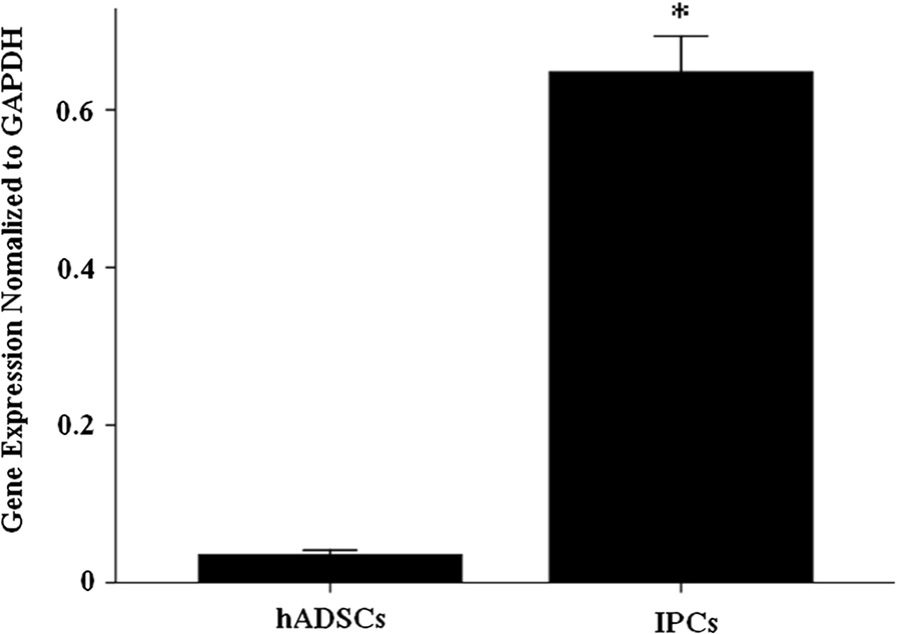
**Gene expression analysis.** Insulin gene expression by two groups of cells was 0.04 ± 0.004 for hADSCs and 0.65 ± 0.036 for IPCs; cycle threshold values of PCR assay were 14.12 ± 0.45 and 14.33 ± 0.37, respectively. Gene expression was normalized to GAPDH. The asterisk denotes *P* < 0.05.

Insulin cannot be used as a differentiating medium, so the insulin that appeared in media after glucose stimulation was synthesized *de novo* and secreted by IPCs. Figure 1 shows that the expression of insulin gene massively increased. Insulin mRNA expression in IPCs increased 16-fold, from day 0 to day 12 (*P* < 0.05). To verify whether IPCs could secrete insulin as a result of sensing physiological glucose concentrations as beta cells do, we first detected the quantity of insulin secretion in different glucose concentrations and under different stimulating time frames. ELISA (Table 2) showed that beta cells and IPCs from all four donors secreted insulin after 30 min or 1 h of stimulation, with no difference existing between 30 min and 1 h of stimulation in high glucose concentrations. However, in low glucose concentrations, the amount of insulin was obviously lower than that in high-glucose stimulation for 30 min or 1 h. Interestingly, normal human pancreatic beta cells responded to low glucose concentrations after 30 min of stimulation, and the amount of insulin was similar to the amount resulting from 1 h of stimulation. On the other hand, IPCs hardly secreted any insulin (0.46 ± 0.04 μU/mL) after low-glucose stimulation for 30 min and only secreted a little insulin (1.01 ± 0.11 μU/mL) after 1 h of stimulation in low glucose concentrations. Our data illustrated that insulin secretion from both normal beta cells and IPCs were regulated by glucose. However, the amount of insulin secreted by beta cells was much higher than that by IPCs (*P* < 0.05).

**Table 2:** Insulin secretion of cells (μU/mL)

	**L-glucose**	**L-glucose**	**H-glucose**	**H-glucose**
	**(30 min)**	**(1 h)**	**(30 min)**	**(1 h)**
Normal human pancreatic β cells	9.25 ± 1.14	9.65 ± 1.12	23.43 ± 4.12	25.81 ± 2.57
IPCs	0.46 ± 0.04	1.01 ± 0.11	1.20 ± 0.13	1.50 ± 0.23

L, low; H, high.

### Morphology of cells as observed by AFM

For each group, two coverslips containing six cells each were analyzed. There was not much difference in appearance between the beta cells and IPCs observed via an inverted microscope. Single-membrane proteins may reveal the details of cell surface structures which can be observed by AFM. Therefore, we analyzed the nanostructures of beta cells and IPCs through AFM in contact mode. IPCs had similar morphological features to beta cells which appeared as polygons, ovals, or circles. IPCs were bigger than beta cells (*P* < 0.05; Table 3).

**Table 3:** Characteristic of cells

	**Normal human pancreatic β cells**	**IPCs**
Length (μm)	55.46 ± 4.84	73.45 ± 2.08*
Width (μm)	34.71 ± 1.57	40.78 ± 1.09*
Height (nm)	505.39 ± 12.01	421.46 ± 19.25*

*Compared with normal human pancreatic β cells, the difference was significant, *P* < 0.05.

**Figure 2:**
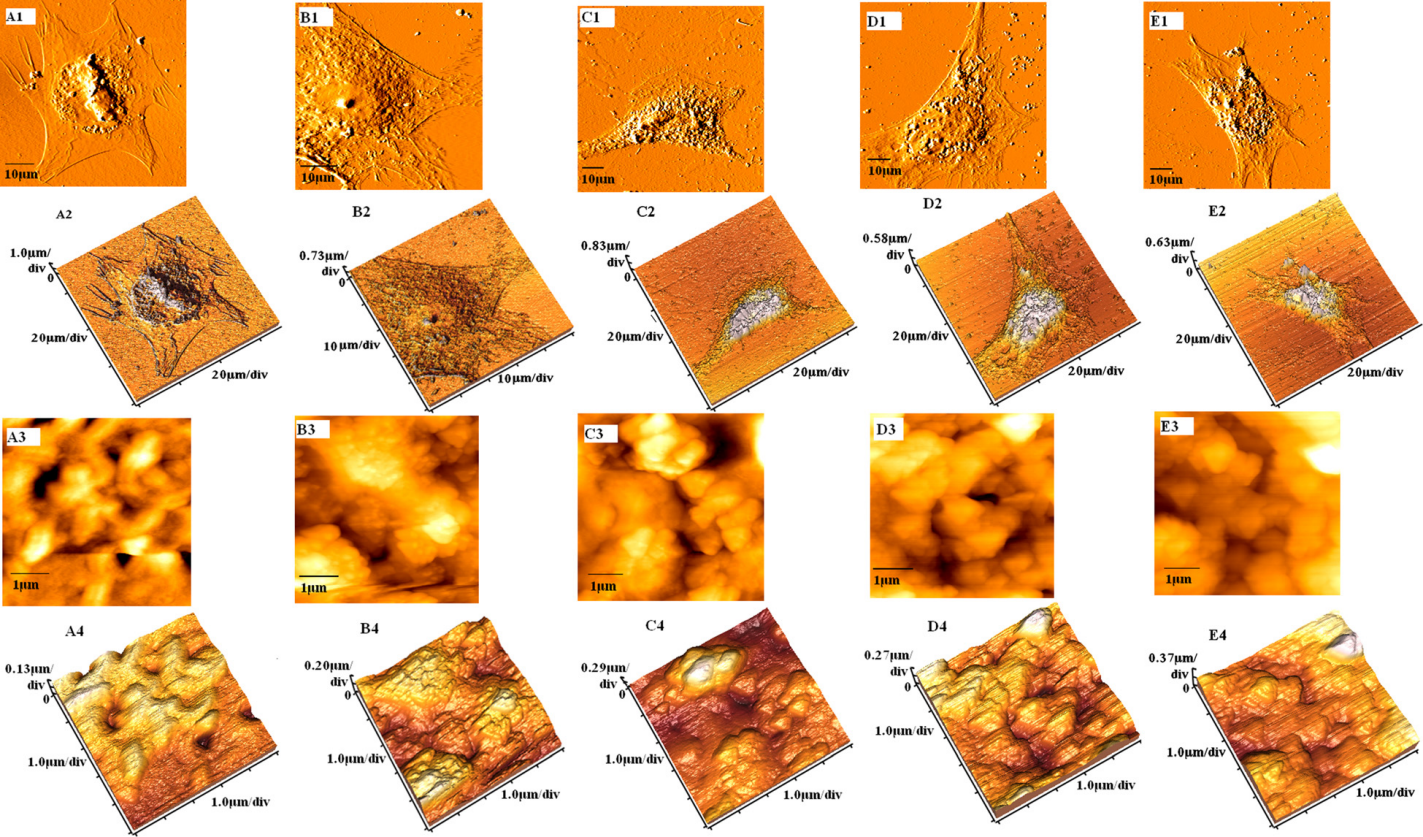
**Morphological changes of human normal pancreatic beta cells, as detected by AFM.** Treated with D-PBS (A1 to A4), high-glucose medium for 1 h (B1 to B4), high-glucose medium for 30 min (C1 to C4), low-glucose medium for 1 (D1 to D4), low-glucose medium for 30 min (E1 to E4). A1, B1, C1, D1, and E1 show the morphology of the whole cell; A3, B3, C3, D3, and E3 show surface ultrastructures on corresponding cells in images A2, B2, C2, D2, and E2; A4, B4, C4, D4, and E4 show 3D structures of the cells.

**Figure 3:**
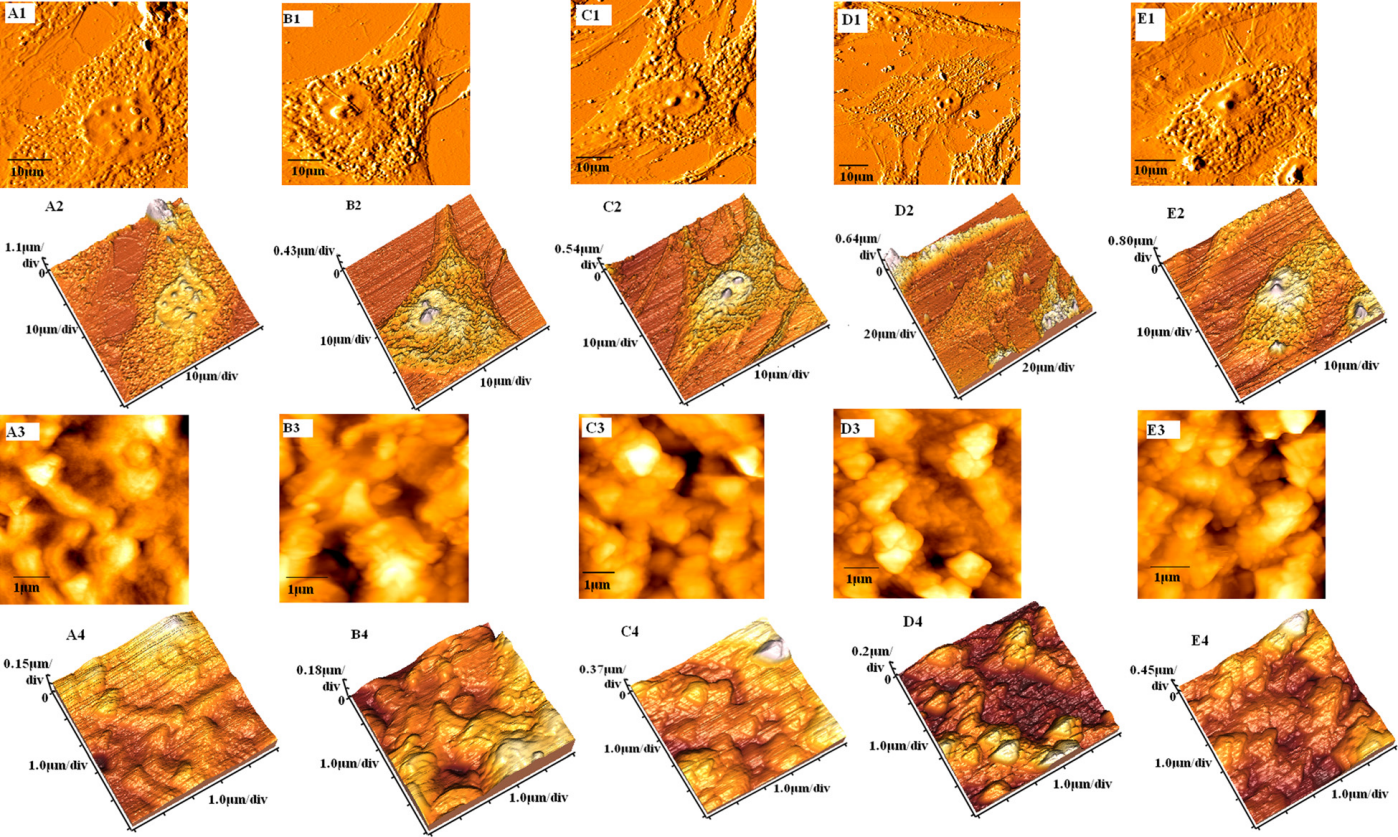
**Morphological changes of IPCs, as detected by AFM.** Treated with D-PBS (A1 to A4), high-glucose medium for 1 h (B1 to B4), high-glucose medium for 30 min (C1 to C4), low-glucose medium for 1 h (D1 to D4), low-glucose medium for 30 min (E1 to E4). A1, B1, C1, D1, and E1 show the morphology of the whole cell; A3, B3, C3, D3, and E3 show surface ultrastructures on corresponding cells in images A2, B2, C2, D2, and E2; A4, B4, C4, D4, and E4 show 3D structures of the cells.

Figures 2 and 3 show a characteristic structure with many holes located in the cytoplasm in beta cells and IPCs. The porous structure was more obvious in the glucose-stimulated group. We measured the Ra in the analytical area. The statistical results showed that the Ra of the beta cells was bigger than that of the IPCs, regardless of whether glucose stimulation was provided (Table 4). We also measured the nanoparticle size of cells through AFM. The data indicate that the nanoparticle size of beta cells was bigger than that of IPCs, regardless of whether they were subject to glucose stimulation. Moreover, for normal human pancreatic beta cells, the Ra values were similar to each other when comparing 30-min stimulation with 1-h stimulation within the same glucose concentration (*P* < 0.05). However, in the IPCs group, Ra values were much lower when cells were stimulated for 30 min by low glucose concentrations, which was similar to the case observed in a non-glucose state (*P* > 0.05). Particle size trends resembled those of the Ra values. Meanwhile, due to the nanometer-scale resolution of AFM, we observed single-membrane proteins and revealed details of the cellular surface structure. Figures 2 (A3) and 3 (A3) showed that the membrane proteins of both beta cells and IPCs exhibited a homogeneous granular distribution. Figures 2 (B1 to B4, C1 to C4, D1 to D4, E1 to E4) and 3 (B1 to B4, C1 to C4, E1 to E4) evidenced that the cell surface architecture of the normal human pancreatic beta cells and IPCs had been changed and membrane proteins exhibited some extent of aggregation after glucose stimulation, except for IPCs stimulated for 30 min in low glucose concentrations (Figure 3 (D1 to D4)).

**Table 4:** Morphological features of three groups of cells

		**Normal human pancreatic β cells**	**IPCs**
Ra (nm)	N-glucose	107.05 ± 10.77	30.50 ± 1.61
	H-glucose (30 min)	135.05 ± 6.46*	41.88 ± 2.38*
	H-glucose (1 h)	138.26 ± 11.76*	49.41 ± 7.42*
	L-glucose (30 min)	115.81 ± 46.86*	30.76 ± 1.29
	L-glucose (1 h)	129.99 ± 15.33*	36.58 ± 2.99*
Particle size (nm)	N-glucose	215 ± 7.9	152 ± 5.7
	H-glucose (30 min)	345 ± 9.35*	225 ± 7.9*
	H-glucose (1 h)	360 ± 8.0*	233 ± 10.4*
	L-glucose (30 min)	221 ± 12.94*	160 ± 7.90
	L-glucose (1 h)	229 ± 14.74*	169 ± 9.62

*Compared with N-glucose, the difference was significant, *P* < 0.05. N, none; H, high; L, low.

### Observation of cytoskeleton in human normal pancreatic beta cells and IPCs

**Figure 4:**
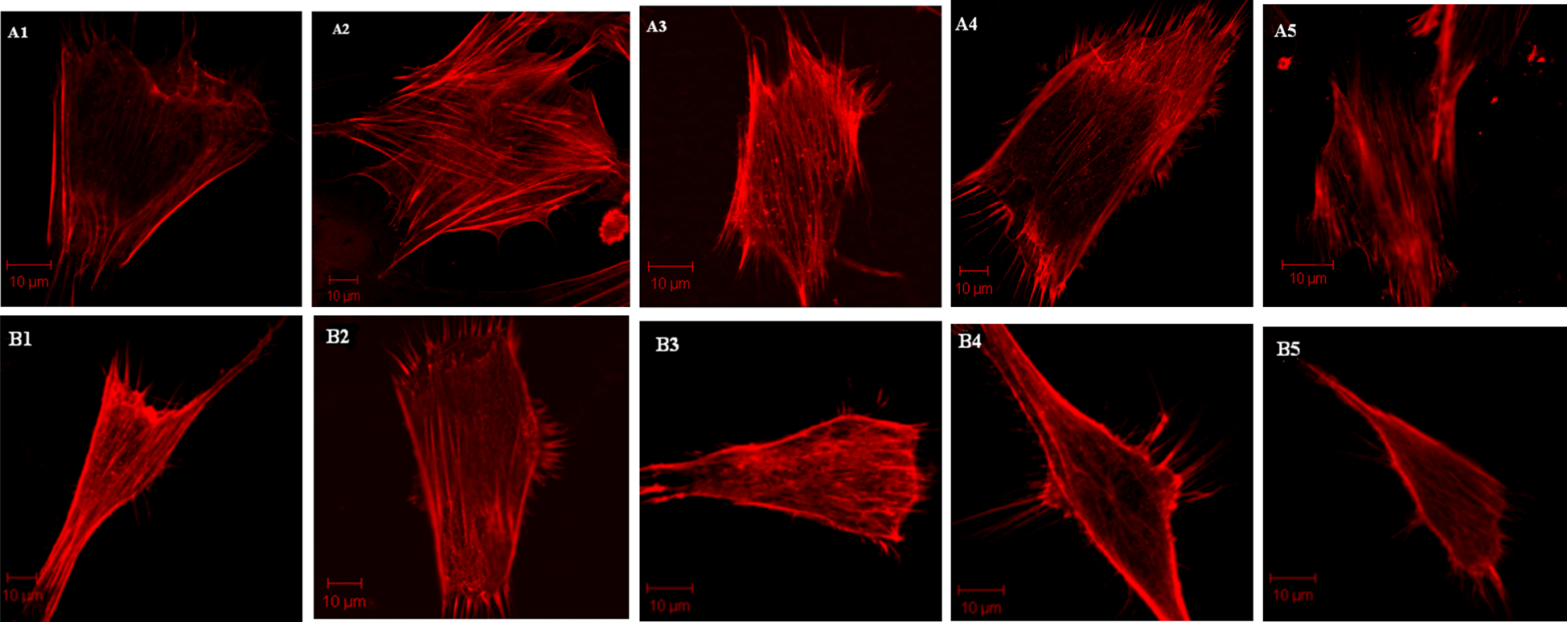
**Distribution of F-actin in normal human pancreatic beta cells and IPCs treated with sugar.** A and B show confocal images obtained using phalloidin-rhodamine to visualize actin in normal human pancreatic beta cells and IPCs. A1 and B1 were treated with D-PBS, the cortical F-actin network of these cells were continuous and dense; A2 and B2 show the cells in high-sugar-stimulated medium after 30 min; A3 and B3 show the cells in high-sugar-stimulated medium after 1 h; A4 and B4 show the cells in low-sugar-stimulated medium after 30 min; A5 and B5 show the cells in low-sugar-stimulated medium after 1 h.

To prove whether exocytosis in IPCs and beta cells was enhanced after glucose stimulation, we analyzed the distribution of the cytoskeleton in these two cell populations. IPCs and beta cells were stained with phalloidin-rhodamine in order to visualize the intracellular actin distribution (Figure 4). When both the beta cells and IPCs were not stimulated with glucose, the F-actin network mainly consisted of parallel, dense, and continuous fibers (Figure 4 (A1, B1)). After 30 min or 1 h of glucose stimulation, regardless of concentration, the subcellular distribution of F-actin in beta cells was sparse and disorganized. However, the cortical actin network did not depolymerize in IPCs after 30 min of low-glucose stimulation (Figure 4 (B4)), but did depolymerize after 1 h of stimulation. Our results showed that the distribution of the cortical actin network in IPCs closely resembled that in beta cells. This process suggested that IPCs might have a similar insulin secretion mechanism as normal beta cells.

## Discussion

We successfully extracted hADSCs from human adipose tissue according to the method reported in the literature [14,15] and characterized the phenotypes of hADSCs through flow cytometry. After that, we used a simple chemical method not involving insulin to differentiate hADSCs into IPCs *in vitro*. In order to assess the function of IPCs, we tested the glucose-induced insulin secretion of IPCs and beta cells *in vitro.* Our data show that regardless of whether they were stimulated for 30 min or 1 h, the beta cells could release a certain amount of insulin after stimulation with high or low glucose concentrations. However, only when stimulated for 1 h in low glucose concentrations did IPCs secrete a little bit of insulin. The results indicate that IPCs can secrete insulin in response to glucose stimulation, similar to, but not as well as beta cells. Even though we only compared beta cells and one kind of IPC which was derived from one source using one differentiation method, our results made evident the difference in physiological function between these IPCs and beta cells. This evidence led to the question: ‘What were the reasons for the difference between IPCs and beta cells?’ We conjectured that these differences were due to the differences in cellular structure. To confirm our hypothesis, we first used AFM to detect cell surface ultrastructure of beta cells and IPCs.

AFM images indicated the changes in morphological properties of IPCs and beta cells stimulated by glucose. The morphologies of IPCs and beta cells were similar to each other, as observed via AFM. They all were polygonal and contained visibly porous features in the cytoplasm. AFM is a common method used to observe cell morphology. However, few studies have reported that these porous structures existed naturally on the cell surface [16-20]. Pores on the cell surface generally appeared after treatment with some drugs [21,22]. Nevertheless, the pores observed after drug treatment were not the same as the porous structures we detected. The porous structures in the IPCs and beta cells were organized and well distributed around the nuclei. The pores that appear after drug treatment are dispersed and isolated. Kim et al. deemed that these isolated holes on the cell surface after drug treatment might be one form of cell apoptosis [22]. Additionally, we speculated that these uniform holes arranged in the cytoplasmic membrane might be dependent onto the type of cells. Beta cells are endocrine cells, while IPCs are artificially synthesized copies of endocrine cells. The pores in the cytoplasmic membrane might be indicative of the exocytosis process through which the hormone is released into the extracellular space.

Simultaneously, we used AFM to compare the cell membrane particle size and Ra of the membrane surface before or after glucose stimulation of IPCs and beta cells. Our results revealed that both membrane particle size and Ra of beta cells were larger than those of IPCs. When both two groups of endocrine cells were stimulated by glucose, the membrane particle size and Ra were higher than those not stimulated, except for IPCs that were stimulated for 30 min with low glucose concentration. The magnitude of cellular Ra, as well as the types, structure, and quantity of membrane protein molecules, directly influenced the inclines and declines of the membrane surface [23]. We speculated that the reason for the lower membrane particle size and Ra in IPCs might be due to their lower membrane protein content. The cell membrane accomplishes its biological function through membrane liquidity, and exocytosis is one of the functions that depend on membrane liquidity [24,25]. IPCs and beta cells secreted insulin through exocytosis. In the meantime, their plasma membranes were replenished via membrane liquidity. We inferred that the change in membrane liquidity might cause the increase in cell membrane particle size and Ra after glucose stimulation.

Beta cells secrete insulin through exocytosis. In beta cells, actin filaments form a dense network under plasma membrane. This actin network acts as a barricade, preventing passive diffusion of insulin follicles to the plasma membrane. Thus, the actin network ultimately lessens insulin secretion via reduction of exocytosis [26]. On the contrary, F-actin depolymerization can increase exocytosis, which increases insulin secretion. We proposed that the pores we observed that were located in the cytoplasmic membrane were one of the characteristics of insulin exocytosis, and increased evidence of porous structures may be related to the enhancement of insulin exocytosis.

To prove that exocytosis had been enhanced after glucose stimulation of IPCs and beta cells, we demonstrated that without glucose stimulation, the actin network underneath the plasma membrane was continuous and dense. After glucose stimulation, the actin network depolymerized and became discontinuous. After F-actin depolymerization, inhibition of exocytosis was relieved and insulin secretion increased. Interestingly, in the IPCs group, the cortical actin network did not depolymerize in low glucose concentrations after 30 min of stimulation. The actin network became discontinuous and depolymerized only after low-glucose stimulation for 1 h.

## Conclusions

In conclusion, our data proved that only normal human pancreatic beta cells could release insulin after low- and high-glucose stimulation for 30 min and 1 h. The cellular ultrastructure and function of IPCs resembled closely those of the normal human pancreatic beta cells. However, there is still so much diversity between the two groups of cells. These differences might provide a future research direction to figure out how to optimize differentiation into IPCs. In our study, we only tested the difference between one kind of IPC and normal human pancreatic beta cells. Therefore, our results are not enough to elucidate the relationship between cellular ultrastructure and function. In order to explore the relationship between cellular structure and cell function, we need to study the links between cell function and more cell membrane proteins, as well as analyze various types of endocrine cells by looking for the common cellular surface ultrastructure.

## Competing interests

The authors declare that they have no competing interests.

## Authors' contributions

QPS and SML carried out the fabrication of samples and the AFM and LSCM measurements and drafted the manuscript. XHL carried out the immunoassays. HYJ performed the molecular genetic studies and participated in the sequence alignment. JYC, LXZ, and LF initiated, planned, and controlled the research process. All authors read and approved the final manuscript.
